# Behavioral pieces of neuroethological puzzles

**DOI:** 10.1007/s00359-016-1143-7

**Published:** 2017-03-04

**Authors:** Kenneth C. Catania

**Affiliations:** Department of Biological Sciences, Vanderbilt University, Box 351634 Station B, Nashville, TN 37235−1634 USA

## Abstract

In this review, I give a first-person account of surprising insights that have come from the behavioral dimension of neuroethological studies in my laboratory. These studies include the early attempts to understand the function of the nose in star-nosed moles and to explore its representation in the neocortex. This led to the discovery of a somatosensory fovea that parallels the visual fovea of primates in several ways. Subsequent experiments to investigate the assumed superiority of star-nosed moles to their relatives when locating food led to the unexpected discovery of stereo olfaction in common moles. The exceptional olfactory abilities of common moles, in turn, helped to explain an unusual bait-collecting technique called “worm-grunting” in the American southeast. Finally, the predatory behavior of tentacled snakes was best understood not by exploring their nervous system, but rather by considering fish nervous systems. These experiences highlight the difficulty of predicting the abilities of animals that have senses foreign to the investigator, and also the rewards of discovering the unexpected.

## Introduction

As an undergraduate in the late 1980s, I first tentatively dipped my toe into the world of research as an assistant at the National Zoo in Washington DC. Part of my job was to conduct behavioral experiments testing whether star-nosed moles had electroreception. The results were disappointing; the moles seemed indifferent to electric fields. However, I went on to study star-nosed moles in graduate school, where I discovered that they have a remarkable somatosensory system and related brain structures. The experience taught me two valuable lessons that have been reinforced many times over the years. First, in the absence of data, it is hard to predict outcomes. Put less formally, exciting ideas rarely pan out. This has been such a pervasive theme in my career that it is tempting to keep a running tally of failed ideas and experiments. It would be a depressing statistic, if not for the second lesson. There is something very interesting about every species, it is just seldom obvious.

Detailed behavioral data are often key to finding the “interesting something” that may not be obvious. This, of course, is a major theme in neuroethology. It is a thread that has run through all of the research in my laboratory. In this review, I have chosen to give a first-person account of how new behavioral data have provided important and often surprising insights about diverse species. Sometimes, this has added a new piece to a complex puzzle; more often, it has revealed an entirely new puzzle. Rarely, the entire puzzle has emerged fully assembled. Here, I recount some of these studies and the insights provided by the behavioral dimension.

### The distorted map

**Fig. 1:**
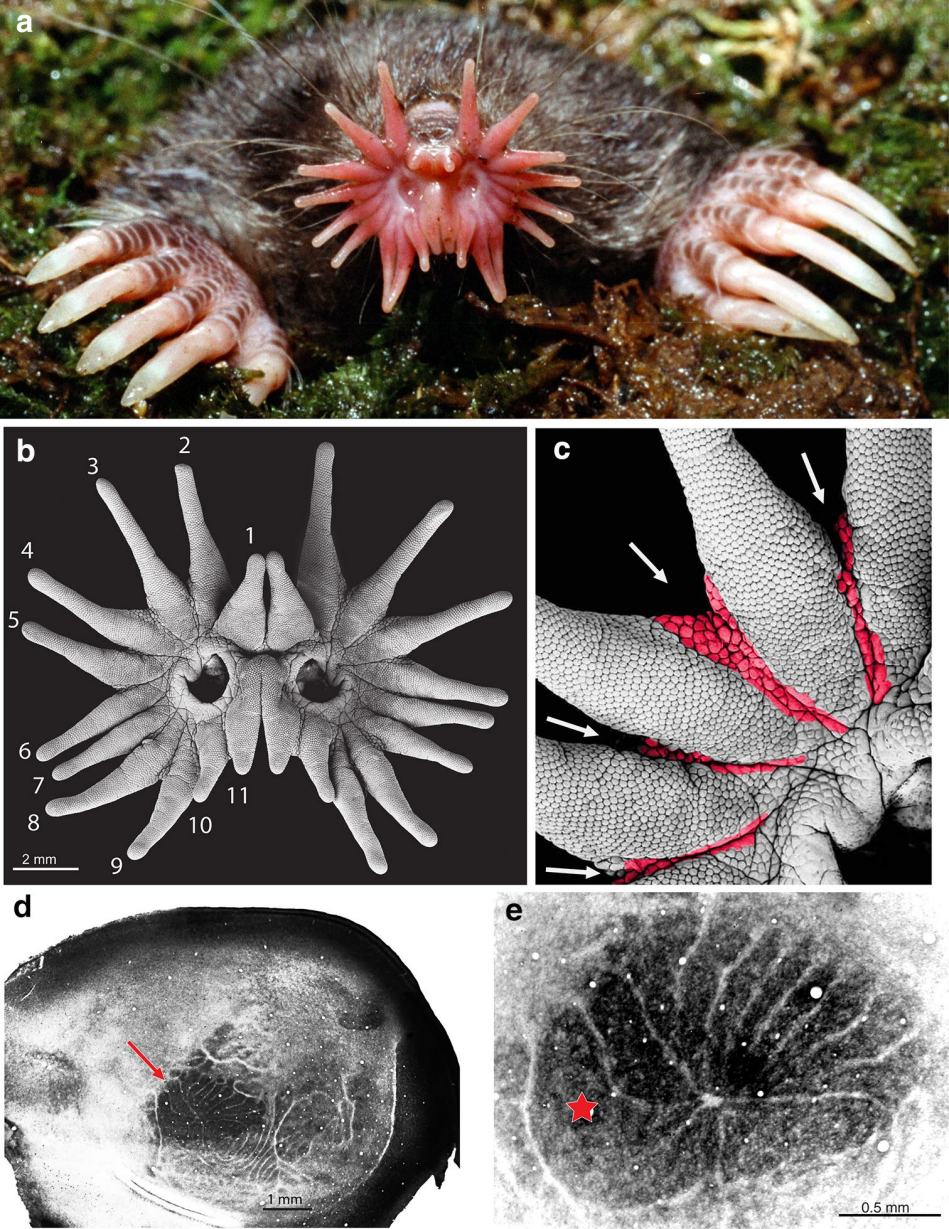
The unusual nose and brain of the star-nosed mole (*Condylura cristata*). **a** Star-nosed mole emerges nose-first from its tunnel, revealing the 22 fleshy appendages (rays) that ring the nostrils. Large clawed forelimbs are used for digging tunnels in North American wetlands. This animal weighs about 40 g. **b** Star under the scanning electron microscope, revealing thousands of domed mechanoreceptors called Eimer’s organs. The nasal rays are numbers from 1 to 11 on each side of the nose starting at the dorsal midline. **c** Closer view of Eimer’s organs on the rays, showing strips of tissue (*colorized*) lacking Eimer’s organs between the rays. **d** Flattened section of layer 4 neocortex processed for the metabolic enzyme cyctochrome oxidase to reveal the visible star representation (*arrow*) in primary somatosensory cortex (S1). **e** Closer view of the visible star representation (different case from **d**) showing the 11 subdivisions that represent the contralateral star. *Red star* marks the unusually large representation for ray 11

Star-nosed moles (*Condylura cristata*) are one of about 30 species of moles that make a living burrowing through soil and feeding on invertebrates. The star-nosed mole has a unique anatomy, and a unique habitat (Yates 1983). It is the only mole that lives in the muddy soil of wetlands, and it is the only mole (and only mammal) with 22 appendages (rays) that ring its nostrils (Van Vleck 1965). As such is has been the subject of curiosity and speculation, since it was first described in the 1800s. In the 1980s, the radar-dish appearance of its nose (Fig. 1) may have added to the impression that it might function as an antenna, perhaps, detecting electric fields as proposed for the bill of the duck-billed platypus (Manger and Pettigrew 1996). This intriguing early hypothesis (Gould et al. 1993) has not been supported by subsequent anatomical or behavioral studies (Catania 2000a). However, while investigating this possibility, I uncovered hints that there might be something special about the star-nosed mole’s brain.

The electrosensory hypothesis motivated me to scrutinize every micron of the star (Catania 1995a, b). Passive electroreceptors generally require some access of nerve endings or hair cells to the environment, or at least a low resistance pathway to a transducing cell (Zakon 1986). There was no evidence for such a configuration on the star. Instead, the star is completely devoted to a single receptor structure, the mechanosensory Eimer’s organ (Fig. 1b). Eimer’s organs are found on all mole species that have been examined, with very rare exception (Catania 2000a). Thus, the star is an elaboration on a common theme among moles. The Eimer’s organs on the star are smaller than those of other species, and the large surface of the star provides room for many more of these organs than found on other mole noses. It would be accurate to describe the star as “made” of Eimer’s organs. As such, it is a remarkable touch organ.

The “hint” that I mentioned about the mole’s brain came in the form of subtle divisions between each star ray (Fig. 1c). Even where the epidermis is continuous between rays, there is a thin strip of tissue devoid of Eimer’s organs that separate the adjacent sensory sheets. This may seem like an insignificant observation, but I was reminded of the separate whiskers on rats and mice, and the corresponding barrels in the neocortex (Woolsey and Van der 1970; Woolsey et al. 1975). Cortical barrels are anatomically visible representations of the whiskers in the primary somatosensory cortex (S1). They provide many advantages for studies of the rodent nervous system in the same way as individually recognizable cells of invertebrates (and some vertebrates-Mauthner cells) but on a different scale. Once the barrel system was mapped by recording from neurons, investigators could conduct a myriad of studies of response properties, connections, development, plasticity, anatomy, and circuit analysis, all while being confident of where they were in the brain and how the area was organized. A visible map of the star in mole cortex would provide all these advantages in a new and very different species.

Searching for an anatomically visible brain map required a combination of electrophysiological recordings and flattening the neocortex to obtain sections of layer 4 that contained most of primary somatosensory cortex (S1). From the earliest experiments, it became clear that star-nosed moles had such a visible map. This was very exciting, but the map was hard to interpret, because it did not seem to match the star. There were clearly visible stripes in S1, with each stripe containing neurons that responded to a single ray. But early on, it was not possible to record from the representation of every ray in each experiment, and it looked from the anatomy as if there where only ten stripes radiating from a large, somewhat u-shaped structure in more lateral cortex (only half of the body is represented in each cortical hemisphere, so 11 stripes corresponding to half the star would be expected). Where was the expected 11th stripe, and what was the giant structure at the base of the cortical stripes? My first thought was the giant, lateral subdivision represented the mouth, and somehow one of the cortical stripes had not shown up clearly in the sections. However, the neuronal recordings suggested that the top stripes represented only the ten larger rays (numbers 1–10) and responses from the 11th ray were more lateral. I should also mention that star-nosed moles have three visible brain maps in total, so the pattern of cortical stripes is complex. The most obvious map (Fig. 1d arrow) is found in S1—and it was explored first (Catania and Kaas 1995).

More detailed neuronal recordings clearly showed that the 11th ray was represented most laterally-its representation was the giant, enigmatic u-shaped structure. This did not make sense, because the 11th ray is small and has few Eimer’s organs on its surface. In the case of rodents, the size of each whisker dictates the size of each cortical barrel (Welker and Van der 1986). What could account for the opposite trend in star-nosed moles?

**Fig. 2:**
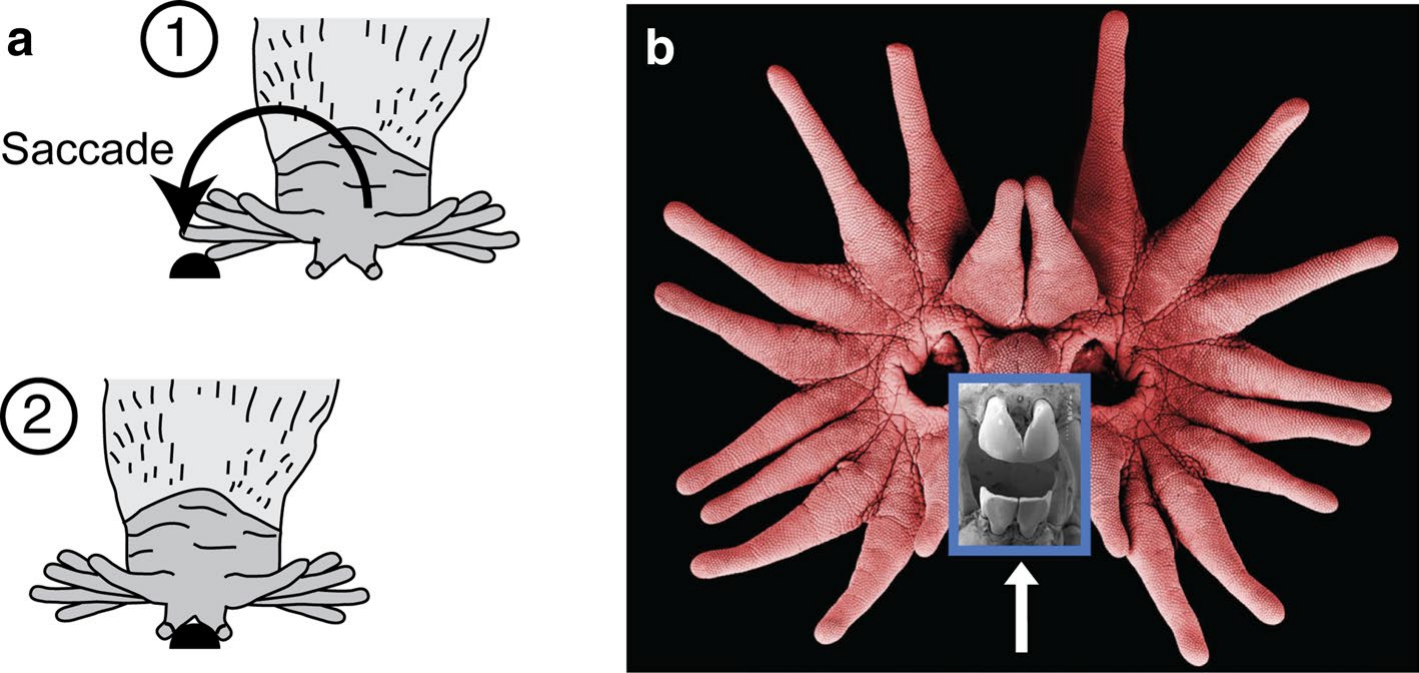
Saccadic star movements. **a** When an object of interest in contacted with the lateral rays (1–10), a sudden movement of the star (lasting about 50 ms) repositions the star for contact with the 11th, foveal rays. **b** Scanning electron micrograph of the *star with an inset* showing the unusual, specialized front teeth (*arrow*) of star-nosed moles, located just behind the somatosensory fovea, and used to efficiently grasp very small prey

The answer to this riddle lay in behavior. Analysis of foraging behavior showed the 11th ray acts as a tactile fovea. It is used for detailed investigations of objects and prey, much as we use our retinal fovea (Catania and Kaas 1997). The star is moved like an eye (Fig. 2) glancing here and there with touch, until it is suddenly shifted in a saccadic (jerky) fashion to move the paired 11th rays onto whatever draws the mole’s attention (usually food). The size of the representation of each ray is closely proportional to the number of touches scored to objects the mole is exploring. And unlike the rodent whisker-barrel system, where each barrel is proportional in size to the number of primary afferents serving the corresponding whisker (Welker and Van der 1986), the behaviorally most important star rays (1, 10, and most especially 11) are magnified in the cortical map far more than would be predicted from the number of afferents serving each ray (Catania and Kaas 1997). The same relationship has been found for the primate visual system, where the representation of the most important foveal ganglion cells is preferentially magnified in primary visual cortex (Azzopardi and Cowey 1993).

As is the case for rodent cortical barrels, the visible maps in the mole’s cortex allowed for many measurements to be made in a clearly defined network (Catania 2011). However, the mole’s behavior also opened unanticipated new doors. Saccadic eye movements in primates are too fast to record with standard video equipment, and the same is true for saccadic star movements. High-speed video was used to record the sequence of touches made to objects with different parts of the star. The initial goal was to document the surprising similarities between star movements and eye movements (Catania and Remple 2004). However, the data revealed something else entirely; star-nosed moles are incredibly fast.

Recordings of mole foraging behavior included the entire sequence of search, detection, saccadic star-movement, and capture of small prey. The time from detection to capture and resumption of search (handling time) is an average of about 230 ms; the shortest was 120 ms (Catania and Remple 2005). This statistic places star-nosed moles as the fastest know mammalian foragers with the shortest prey handling time (and earned them a place in the Guinness Book of World Records). When this result is put in the context of classical foraging theory (Stephens and Krebs 1986), a large piece of the longstanding puzzle about star-nosed moles fell into place. Short handling times allow star-nosed moles to specialize on small prey for at least part of their diet. These kinds of prey are particularly abundant in wetlands (Anderson and Smith 2000). Specializing on small prey also requires a high-resolution sensory system with sufficient surface area to increase the probability of contacting and detecting prey, and small receptive fields to guide precise orientation movements of the mouth. These are all features of the star. Examination of star-nosed mole dentition (Fig. 2) and previous gut-content analysis (Hamilton 1931) strongly support this interpretation that the star is used for the rapid detection of small prey. Thus, an investigation that began with a search for anatomical clues to electroreception ultimately led through the central nervous system, to behavior, and finally to ecological theories of optimal predator choice (Stephens and Krebs 1986). The behavioral dimension of these studies cracked the case, revealing why the cortical maps are distorted and providing the data that best explained the function of the star and the likely selective advantages that led to its evolution.

### Underestimating the “Common” mole

Star-nosed moles exhibit extremes in anatomy, brain organization, and behavior. This allows them to forage with unparalleled speed and take advantage of small prey. I enjoy showing movies of a star-nosed mole feeding, which never fail to impress an audience. In my favorite clip, a star-nosed mole finds and eats eight different prey in under 3 s. How do other moles fair in comparison? The common, eastern American mole (*Scalopus aquaticus*) lives in dryer, more abrasive soil, and has no star. It has fewer somatosensory maps in the neocortex (Catania 2000b) and is one of the only moles without mechanosensory Eimer’s organs on its small snout (Catania 1995b). It stands out as the mole least specialized for touch (Catania 2000a). This seemed like the ideal species for a comparative foraging “race”. I designed long Plexiglas foraging chambers to record the eastern mole’s behavior. I actually felt a little guilty, because the race seemed rigged; I knew which horse was faster. All I had to do was sit back and watch the poor, starless common mole fumble about its tunnel looking for small prey, and I would have a nice comparative study demonstrating the superior foraging ability of star-nosed moles.

In some ways, this was true. In contrast to star-nosed moles, common moles could not find and eat very small prey. To level the playing field, I increased the size of prey (earthworm segments). With this modification, I began watching common moles searching the tunnel, expecting relatively poor performance. Instead, the common moles moved in a nearly straight line from one prey item to the next, as if they knew precisely where each was located (common moles have tiny eyes hidden below the fur, and the optic nerve is nearly invisible even with a surgical microscope). When they did on occasion pass by prey in the tunnel, they immediately turned back and found it. It was an impressive and unexpected performance. The data showed that what looked like continuous sniffing behavior, suggesting that common moles were depending on olfaction. It was quite different from star-nosed mole behavior, so I was trying to compare apples and oranges.

**Fig. 3:**
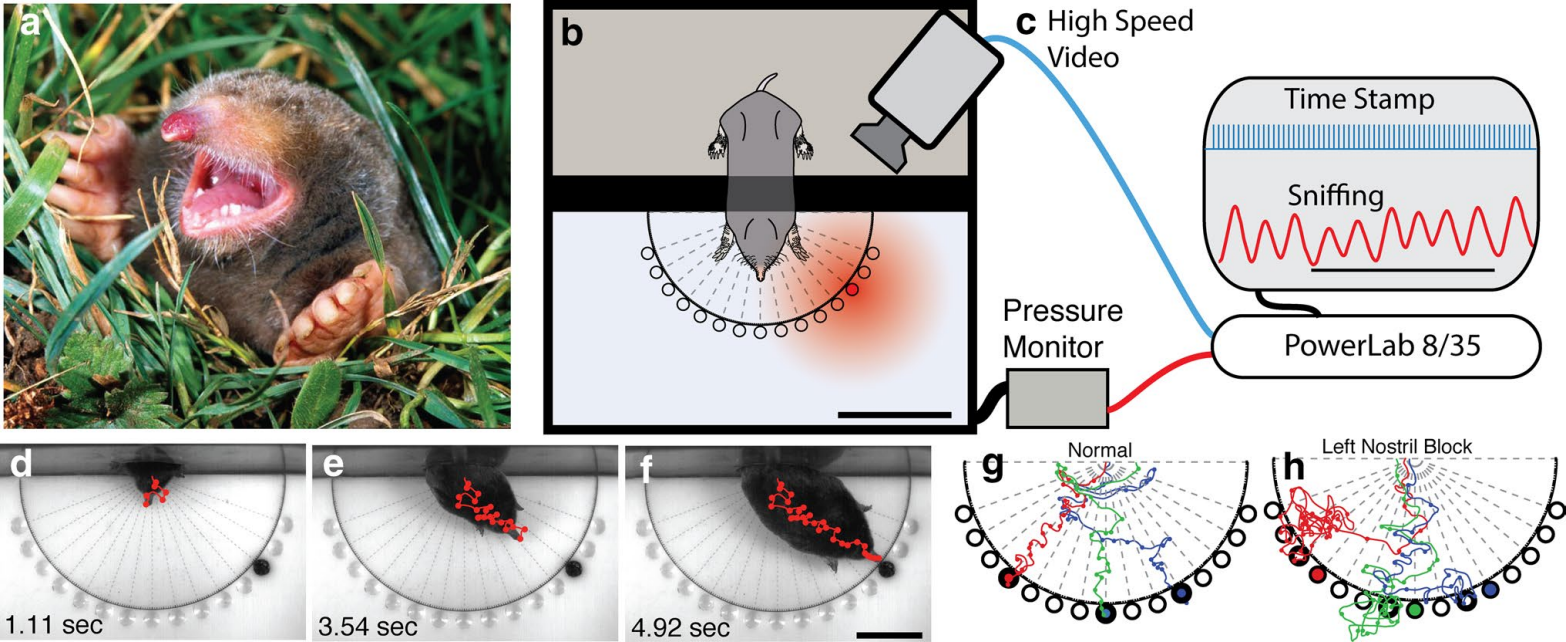
Sniffing and olfactory localization in the common, eastern American mole (*Scalopus aquaticus*). **a** Common mole, lacking both star and Eimer’s organs, emerging from its tunnel. **b, c** Schematic of the chamber and recording paradigm for olfactory localization trials. A temporarily sealed chamber with a pressure monitor was used to record sniffing and high-speed video as the moles located an earthworm segment located in a randomly chosen well. **d**–**f** Nose track (*red line*) with sniffs (*circles*) indicated as a mole moves directly toward the odor source. **g** Examples of tracks and sniffs under normal conditions for three trials. **h** Examples of nose tracks and sniffs for three trials with a left nostril block. Note that the animal is consistently biased toward the open, right nostril

I changed the paradigm to specifically investigate olfactory localization in common moles (Catania 2013). Instead of using widely distributed prey in a tunnel, a chamber with radially positioned wells was used in combination with high-speed video and a pressure monitor that recorded the mole’s respiration, and hence its sniffing (Fig. 3). The latter was possible, because the chamber could be made airtight during trials. Between trials, the chamber was cleaned, and an earthworm segment was placed into a random well. Using this paradigm, the performance of the common mole was recorded and quantified in detail, and the results verified the impression from preliminary trials in tunnels. The moles emerged from the holding chamber, and while moving their snout to and fro in coordination with sniffs, they oriented in the direction of the food and then moved toward their goal, often in a nearly straight line (e.g., Fig. 3d–g). Importantly, there was no scent trail in these experiments. The behavior was so strikingly accurate that I repeated trials under 940 nm infrared lighting (with the same result), so there could be no question about vision (see movies in Catania 2013 for full sets of trials from different animals). I have never seen a more impressive use of olfaction.

After documenting this ability in a number of moles, I began to consider something that I had always thought impossible. What if these animals could compare the intensity of odorants between the two nostrils, essentially sniffing in stereo? Clearly, common moles are using serial sampling of different areas in space (e.g., nose movements coordinated with sniffs), but the addition of stereo cues might further refine localization abilities, especially near odorants where concentration gradients are steep (Louis et al. 2008). The obvious test for this ability was a temporary nostril plug, a paradigm similar to the classic experiments investigating auditory localization in barn owls (Knudsen and Konishi 1979, 1980).

**Fig. 4:**
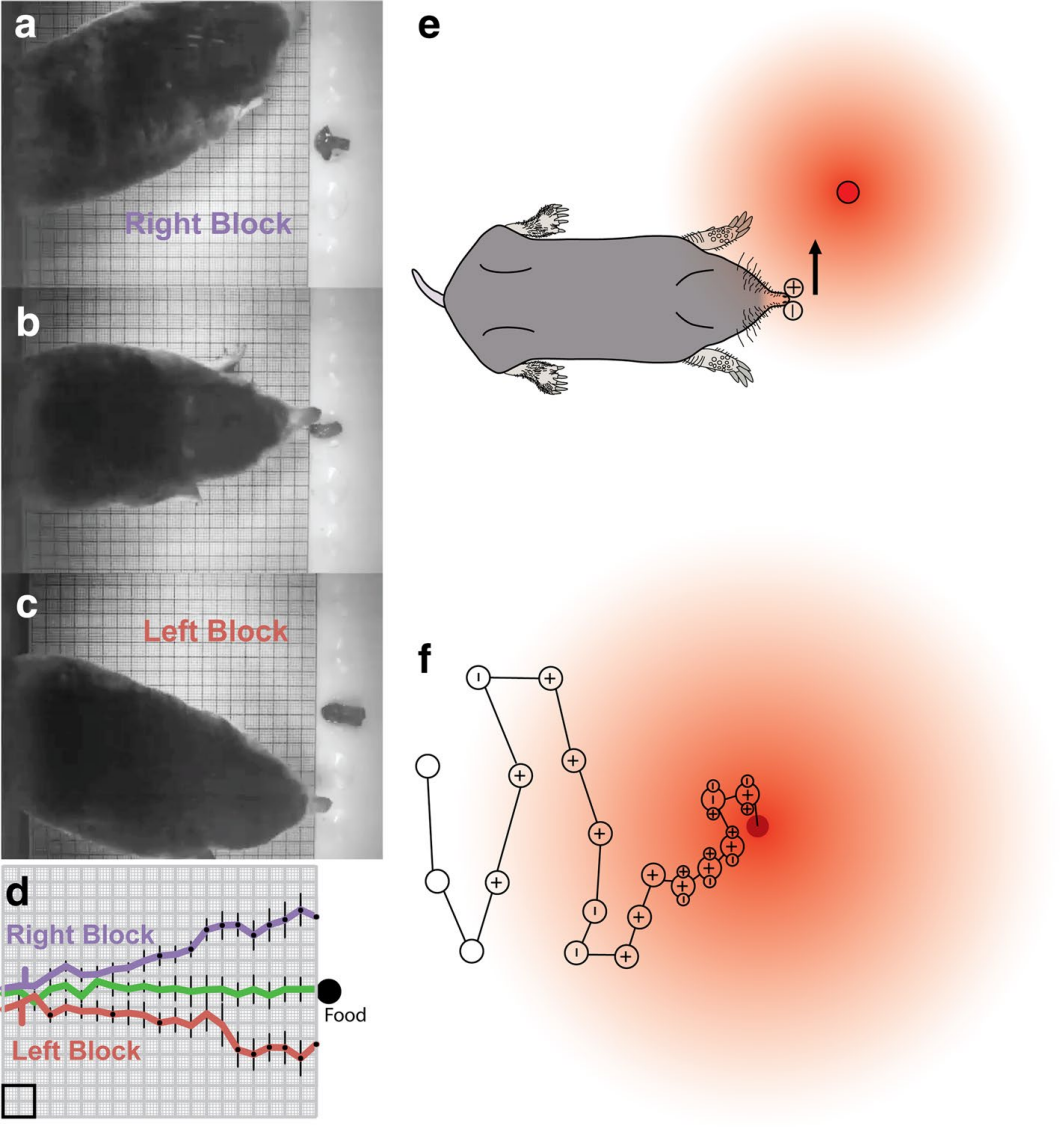
Sniffing and olfactory localization in the common mole. **a**–**c** In this paradigm, the odor source (earthworm segment) was always located in the same location. The mole was tested with right nostril block (**a**), open tube control (**b**), and left nostril block (**c**). **d** Average path for ten trials of each condition, showing the consistent bias in the direction of the open nostril as the mole approached the food. Note, however, that despite the bias and less direct track with nostril block, moles always located the odor source shortly after reaching the end of the grid (not shown, but see Fig. 3h) *Square* 1 cm. **e** Schematic of stereo sniffing, showing “pull” toward the open nostril (+) when odor gradient is steep. **f** Hypothesized use of serial and stereo cues on a path toward the odorant. In this proposal, serial sniffing and nose movements provide the dominant cue (and only cue at a distance), whereas stereo cues refine localization at short distances

I expected this experiment to fail, perhaps, disrupting the mole’s behavior in an ambiguous way, or having no effect. However, the results of nostril plug experiments were quite clear. When one nostril was plugged, common moles were consistently biased in the direction of the open nostril. Figure 3 shows the result for several trials, indicating with lines the path taken by a mole under normal and blocked nostril conditions. When the left nostril was blocked, moles were biased to the right and usually explored the well to the right side of the food first. The opposite occurred for right nostril block, and an open tube used as a control condition demonstrated that blocking airflow was the key variable. Figure 4a–c shows the same effect in a different paradigm during which the food was kept in the same location across different trials, allowing for a more graphical representation of the result (Fig. 4d).

Although the nostril block result was very clear, the effect was limited (Catania 2013). For example, the moles did not turn in circles, and despite taking a less direct route, they still located the food in every trial (Fig. 3h). This suggests, as might be expected, that serial sampling of odorants at different locations, based on large head and nose movements, provides the most important and predominant cue, whereas stereo cues between nostrils play an important role when the mole is close to the food and the olfactory gradient is steep. Figure 4e, f illustrates the suggested roles of serial and stereo sampling at different distances from an odor source.

These surprising results for common moles emphasize the difficulty predicting how different species may be specialized (or not) and also suggest that both star-nosed moles and common moles integrate olfaction and touch while foraging. The relative roles of the two senses for each species will require more complex experimental paradigms, but it is safe to say that eastern moles are olfactory experts. The efficiency of common moles homing in on earthworms adds a potentially important piece to another story, namely, why earthworms must take extreme measure to avoid moles.

### Worm-grunting

**Fig. 5:**
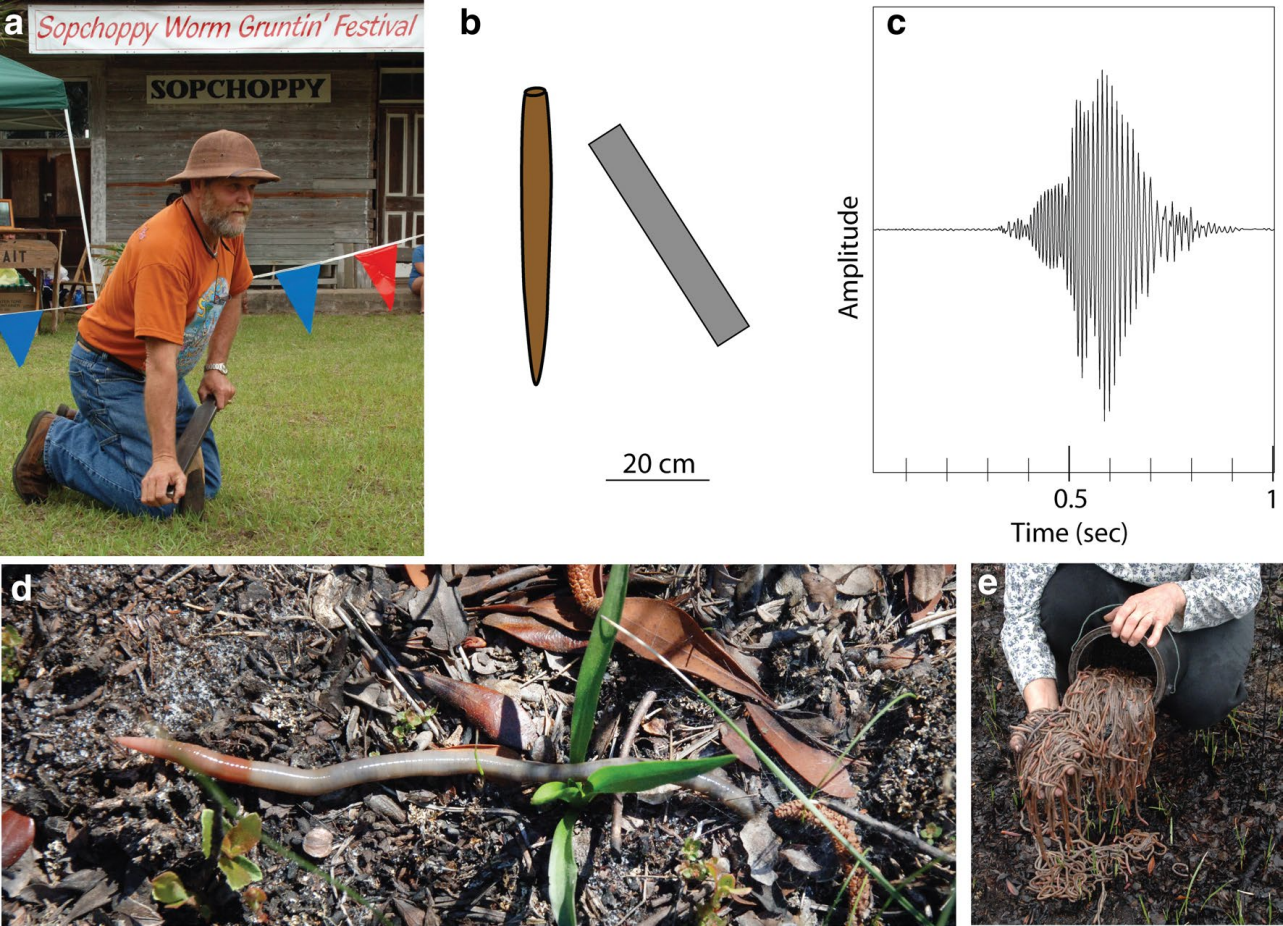
Grunting for worms to collect bait. **a** Gary Revell demonstrates the technique at the annual Worm-Grunting Festival in Sopchoppy Florida. **b** Approximate sizes of the wooden stake and iron strip used during worm-grunting, though preferences vary among worm-grunters. **c** Recording from a vertical geophone place 5 m from the grunter during a single worm-grunting “note”. **d** Diplocardia worm exiting its burrow in response to worm-grunting. **e** Audrey Revell shows the results of just two stake placements (roughly 500 worms)

I suggested at the beginning of this review that exciting ideas rarely pan out (at least for me). But sometimes the first hypothesis is actually correct. The odds of this are probably greatly improved if Charles Darwin had the same idea. In this case, I am talking about an unusual bait-collecting technique called “worm-grunting” that has been practiced the southeastern United States for many decades (perhaps centuries). The practice consists of pounding a wooden stake into the ground and rubbing it with a piece of thick iron to generate strong vibrations that propagate through the ground for many meters. If this is done (properly) in or around the Apalachicola National Forest, hundreds of large, native earthworms will emerge from their tunnels and begin to crawl rapidly across the soil surface, where they can be easily collected (Fig. 5). This bait collection technique had its heyday in the 1960s and 1970s, when it was reportedly a multimillion dollar industry (Kaufmann 1986). Little attention was paid to the practice until it was featured in a news story by Charles Kuralt, for his “On the Road” series (Kuralt 1985; Tobin 2002). The news attracted the attention of the federal government, which began taxing the income generated by worm-grunters and regulated the practice by requiring yearly worm-grunting permits.

It is clear from this legacy of bait-collecting that large earthworms (*Diplocardia mississippiensis*) native to the area have a very strong aversion to vibrations, and respond by rapidly exiting their burrows and crawling across the soil surface. But why would they do this, exposing themselves to opportunistic predation, desiccation, and worm-grunters? The earliest clue comes from Charles Darwin who stated “It has often been said that if the ground is beaten or otherwise made to tremble, worms believe that they are pursued by a mole and leave their burrows” (Darwin 1881). Darwin performed a few crude experiments, but never succeeded in confirming the idea.

Given my experience collecting moles and interest in their different foraging tactics, this was a mystery that I was eager to explore (Catania 2008). Were Darwin’s musings correct? In 2007, I accompanied Gary Revell, while he grunted for worms. Gary is one of the few remaining worm-grunters and he knows the Apalachicola National Forest like the back of his hand. He not only sells bait, but also educates people about the technique and local history at the annual Worm-Grunting Festival in Sopchoppy (Fig. 5a). As Gary and his wife collected worms, I surveyed the area for the common, eastern American mole—the only species native to Florida (and the Southeastern United States generally). It turned out that there was a very large population of moles throughout the Apalachicola National Forest, overlapping every worm collection site that I examined (Catania 2008). The extensive overlap of *Diplocardia* worms and common moles suggested that *Diplocardia* earthworms had been on the mole’s menu during a long, shared evolutionary history. This was a good sign for the hypothesis that worms have an escape response from moles.

The other most obvious explanation was rain. In some parts of the world, earthworms are observed on the soil surface after a downpour. The effect of rain was tested in a number of experiments. Diplocardia worms were collected and housed in outdoor containers and then exposed to long periods of simulated heavy rain until the soil was completely saturated. This had a little effect, in five separate trials, only three out of 250 worms emerged, and the emergence was after more than 15 minutes. Larger containers of earthworms were then observed during real thunderstorms, with a similar result—only 6 out of 900 worms emerged after 25 min of heavy rain. Worms in the soil at the end of these experiments appeared healthy. Finally, the Apalachicola National Forest was surveyed during rainstorms, but no worms were found on the surface.

These experiments suggested that worm-grunters were not simulating vibrations produced by rain, and in retrospect, this seemed obvious. Some species of earthworms are apparently in danger of drowning during heavy downpours (Chuang and Chen 2008), but these species do not bolt to the surface as the first drops begin to fall—instead, it takes many hours. During worm-grunting, worms emerge from their tunnels immediately, in daylight, and often onto dry, hot, sandy soil (sandy soil is characteristic of the area). It would be surprising if vibrations simulating rain superseded all other obvious cues of moisture in the environment. In addition, a strong worm response to rain would probably have changed bait-collecting tactics; stakes and irons could be replaced with raincoats.

Earthworms that emerge during worm-grunting come out of their tunnels at full (worm) speed, and travel across the soil surface before burrowing back down in a process that took an average of more than half an hour. During this dangerous time, some worms were attacked and killed by ants, beetles, or lizards, whereas others (on the hottest days) died from desiccation before they could burrow back into the soil (Catania 2008). These observations suggested worms had a compelling reason to emerge.

**Fig. 6:**
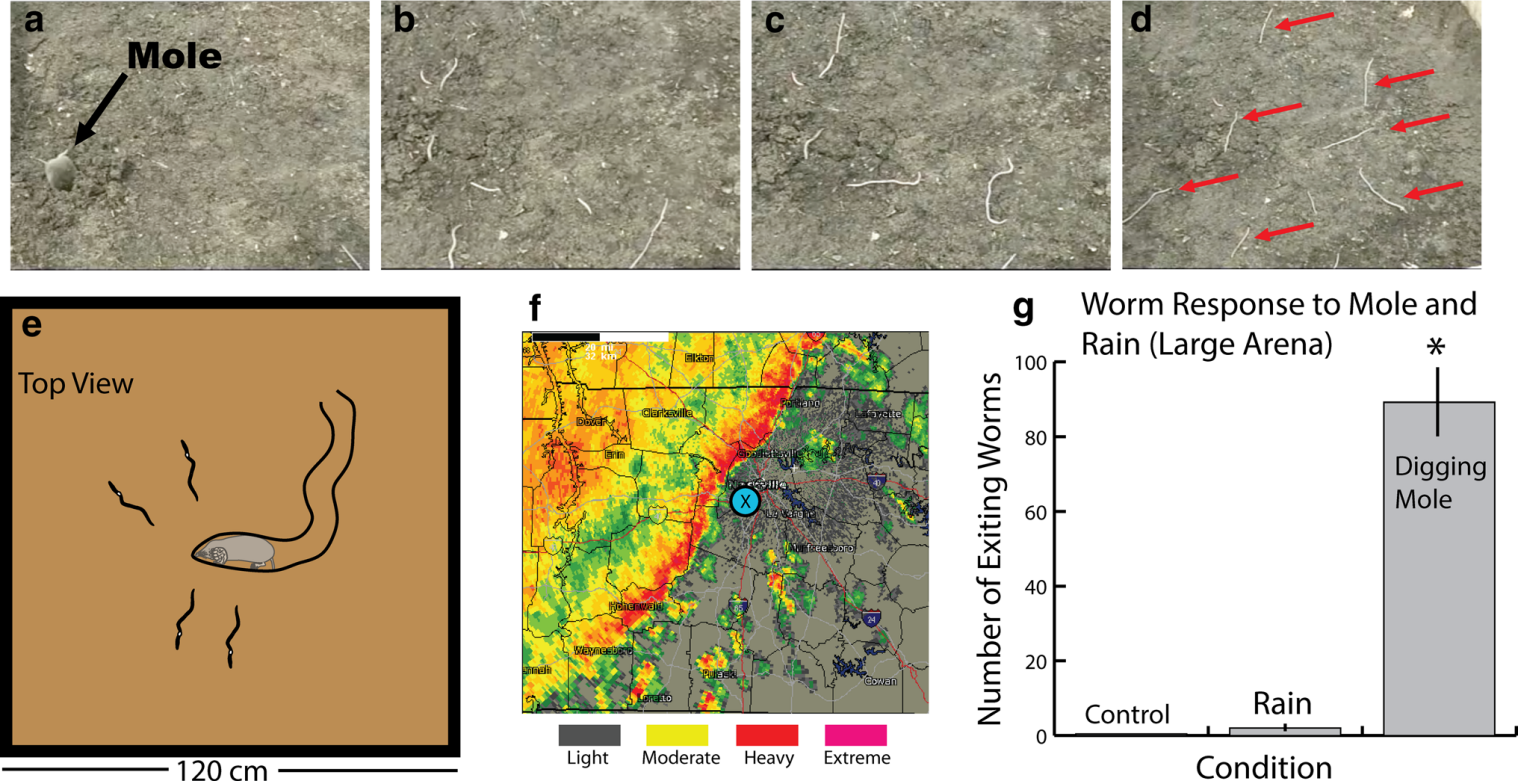
Worm and moles. **a**–**d** Frames captured from video showing the results of a mole (*arrow* in **a**) entering a bin containing worms. Many worms exit to flee across the soil surface (*arrows* in **d**). **e** Schematic of the result. **f** Radar trace just prior to observation of the effect of rain on worm behavior; 300 worms were confined to an outdoor, soil-filled bin. **g** Summary of the results, illustrating the relative effect of rain versus a digging mole

Of course, the most obvious and informative experiment was to put a mole in with the earthworms and observe the results. It was gratifying to perform a first, preliminary version of this experiment in front of Gary Revell. I had caught a common mole, and he had a bucket of soil containing diplocardia worms. We could not resist putting the two together. As the mole dug down into the soil, dozens of worms poured out onto the surface, doing what looked like the worm version of running. The behavior had an uncanny resemblance to worm-grunting responses, and Gary was convinced of the mole explanation. The preliminary test was followed by many controlled experiments measuring worm responses to moles in more natural settings (Fig. 6a–d), analyzing the power spectrum of vibrations from digging moles and worm-grunters (which overlapped extensively in frequency), recording moles digging with geophones and playing back the recordings to worms (which elicited escapes), and noting worm escape responses from wild, foraging moles. All of these experiments pointed uniformly to the conclusion that worm-grunters are unknowingly mimicking the vibrations generated by foraging moles to elicit escape responses (Catania 2008).

When this research was published, the story seemed fairly complete. Moles eat earthworms and earthworms escape from moles. However, the olfactory abilities of common moles add an important piece to the puzzle. Common moles are not simply digging at random, hoping to encounter an earthworm. They have a keen sense of smell and can localize odorants, especially earthworm scent, with unprecedented accuracy. They can also dig tunnels much faster than earthworms. This dynamic is similar to the well-documented arms race between bats and moths (Roeder 1998). Bats can eat a tremendous number of insects, which they detect from a distance. Bats fly much faster than their prey, but echolocation calls propagate well beyond the bat’s detection distance, signaling insects of their approach. Many flying insects have evolved ears to detect bats and engage in drastic evasive maneuvers when loud ultrasound is detected. Similarly, it seems likely that moles detect earthworms scent trails and begin pursuit from a distance. Like bats, moles inevitably provide their prey with a signal of their approach. This comes in the form of vibrations propagated through the soil as they dig. These vibrations are so strong that I discovered and captured several of the moles in the worm-grunting study after hearing them dig (as they broke networks of small roots that are ubiquitous in their habitat—see Catania 2008 for video and sound of digging moles). Given the common mole’s olfactory abilities, a nearby earthworm may be doomed if it stays in place. Escaping through the soil may have equally dire consequences, unless the worm’s tunnel has (literally) no dead ends.

Exiting to the soil surface is a sure way of escaping a common mole, because moles do not pursue prey above ground. The likely reasons for this suggest a twist to Dawkin’s “Life Dinner Principle” which invokes the relative costs of failure for a rabbit and a fox (Dawkins and Krebs 1979). There is more selective pressure on the rabbit to escape death, than on the fox to catch a meal. Similarly, escaping to the surface to avoid death is presumably worth the risk for a worm, but chasing dinner above ground is not worth it for a mole.

Worm-grunting provides a more direct example of a different phenomenon, Dawkin’s “Rare Enemy Effect”, which posits that uncommon predators may take advantage of prey behavior by exploiting behaviors that are usually adaptive (Dawkin’s 1982). In this case, emerging from the soil in response to vibrations is presumably the best strategy in a forest populated with moles, and it is the unlucky worm that encounters a worm-grunter and ends up on a fishhook.

### Scared to death

The last example I want to describe circles back to my undergraduate days working at the National Zoo in Washington DC. In the 1990s I had friends that still worked at the Zoo and I would occasionally visit to catch up and hear about the latest additions to their menagerie. On one of these trips, I was making my usual rounds through the Reptile House, when I noticed the new tentacle snake exhibit. Tentacled snakes (*Erpeton tentaculatum*) are fully aquatic, give live birth in the water, and have a pair of appendages protruding from their upper jaw near the nostrils (in roughly the same location as the heat sensing pits in vipers). It is the only snake with such appendages, and it feeds exclusively on fish; hence, it is other common name, “the fishing snake” (Murphy 2007).

Although this species could hardly be more distantly related to star-nosed moles, there were many parallels. It was a one-of-a-kind, poorly understood, outlier. It was also a specialist predator. And, of course, the nasal appendages were superficially similar to mole nasal rays. I made a mental note of this species, and when I had the opportunity to acquire specimens more than a decade later, I could not resist investigating the mystery.

Although there was speculation about how the tentacles function (Günther 1864; Smith 1943; Shaw 1965; Bellairs 1970; Hahn 1973), a little experimental work had been done. I planned a multi-tiered study of the brain and peripheral nerves combining anatomy with electrophysiological recordings of neurons from tentacle afferents and the optic tectum. However, I started by making some behavioral observations that might help guide experimental design and subsequent interpretations. It would presumably be easy, for example, to determine whether the tentacles act as lures (reminiscent of the alligator snapping turtle’s worm-imitating tongue; Drummond and Gordon 1979) with some simple observations of hunting snakes. I also knew from just-published work by John Murphy (2007), the world’s expert on “homalopsid snakes” (to which the tentacled snake belongs) that tentacled snakes were very efficient hunters. He videotaped the snakes hunting at 30 frames per second and reported “….prey handling time was very short or non-existent. On some of the successful strikes, the fish disappeared within a single frame” (Murphy 2007).   That is astounding—a single frame of normal video is only 33 ms. Could this species eat faster than a star-nosed mole?

**Fig. 7:**
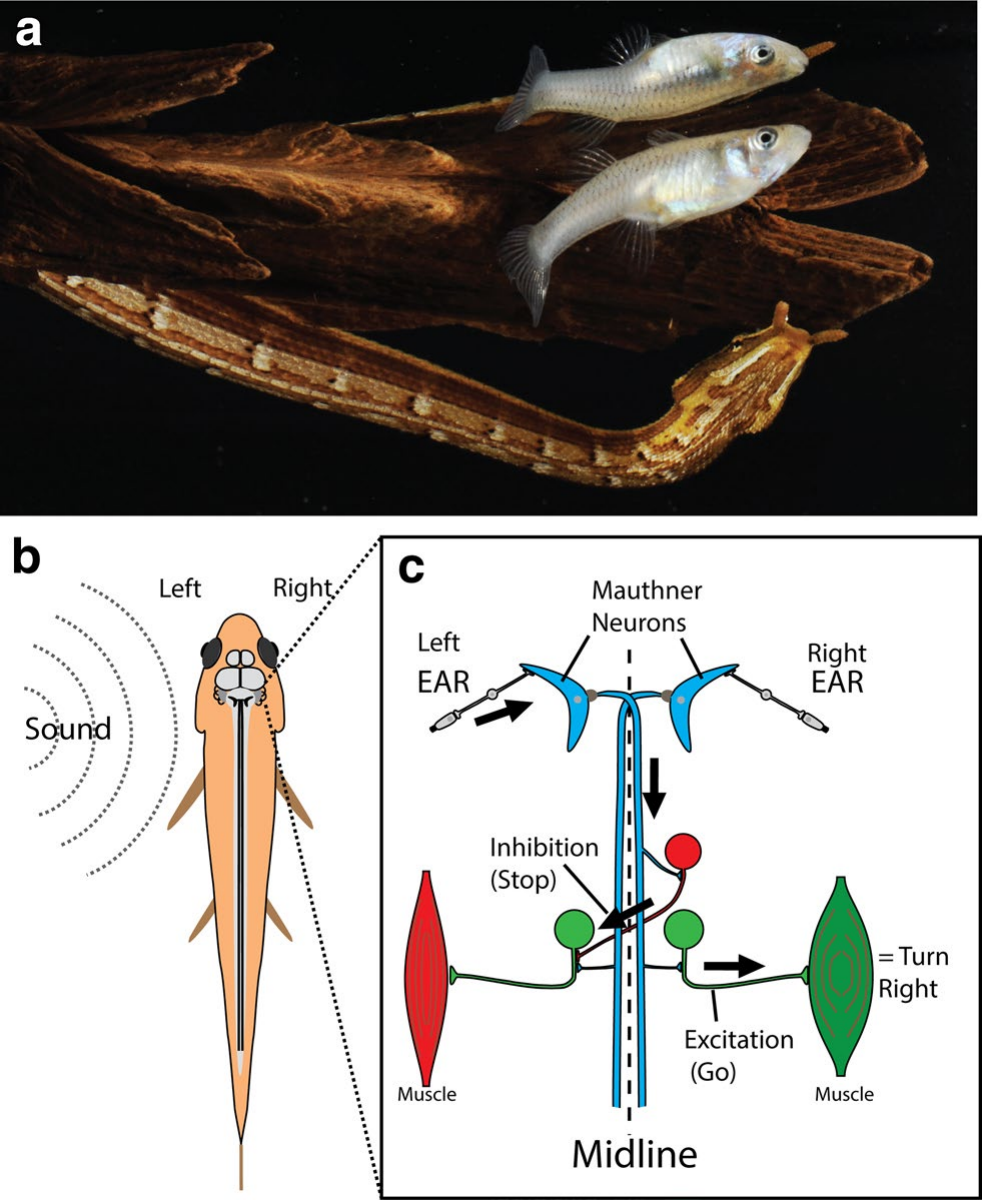
The tentacled “fishing snake” and its prey. **a** Tentacled snake (*Erpeton tentaculatus*) waits for a fish to approach in its characteristic J-shaped hunting posture. **b** Simplified schematic of the neural circuitry that triggers a C-start escape response. In addition, auditory cue arriving on the left side usually generates an action potential in the left Mauthner cell. The transmitting axon crosses the midline to excite motor neurons (*green*) on the right side. Crossed inhibitory neurons ensure muscles on the same side as the stimulus are inactivated

To find out, I began recording snake behavior at 2000 frames per second—the maximal rate of my high-speed camera. Tentacled snakes are sit-and-wait predators with a characteristic hunting posture. They bend their neck (either to the left or the right) to form a J shape (Fig. 7a) and then lie motionless until fish approach. This posture makes them difficult to see when housed with plants and branches typical of their natural habitat (ponds and steams in Thailand, Cambodia, and South Vietnam). It was soon obvious that the tentacles are not lures; fish rarely approached the tentacles (which remain motionless); and snakes did not strike at fish near the tentacles. Instead, nearly all strikes were initiated when fish had entered the concave space between the snake’s head and body. The snakes were patient, allowing many fish to pass closely by, until one entered the “sweet spot”. Then, they struck with incredible speed and they somehow swallowed some fish in only 30 ms (as suggested by Murphy). Most others were caught by the head and partly swallowed during the strike. The “somehow” by which fish where captured and eaten stands out as one of the most devious predator stunts on record (Catania 2009).

To fully appreciate the snake’s tactic, some background on fish escape responses is needed. The neural circuitry controlling fish escape (the C-start) is, perhaps, the best understood among vertebrates, in part because it is mediated by a pair of giant neurons (Mauthner cells) that can be readily identified in individual fish (Zottoli 1977; Eaton et al. 1977; Faber and Korn 1978; Eaton and Hackett 1984; Canfield and Eaton 1990; Zottoli and Faber 2000; Korn and Faber 2005; Faber at al. 2006; Preuss et al. 2006). The C-start can be triggered by sound and water disturbances that activate fish ears and the lateral line system. These inputs synapse onto the large dendrite of a giant Mauthner neuron (one on each side of the brainstem) that sends an axon across the midline to activate motor neurons on the opposite side of the fish body (Fig. 7). Thus, activation of the Mauthner cell on the same side as the attacking predator triggers rapid contraction of trunk muscles on the opposite side and hence the bend away from the threat, which is followed by a tail flip that projects the fish further away. A key part of this circuit includes inhibitory cells that block activation of trunk muscles on the same side as the attack and so prevent the disastrous consequences of simultaneous contraction of both sets of trunk muscles, either from ongoing behaviors or from activation of the other Mauthner cell. This system includes a large, fast-conducting axon and some electrical synapses, allowing small fish to begin their turn in as little as 6 ms after the shock wave from a predatory strike is detected. Within 25 ms, the fish is fully bent into the C-shape—the first stage of the escape sequence (times refer to fathead minnows, *Pimephales promelas*, and Gambusia, *Gambusia affinis*).

Tentacled snakes, on the other hand, have the advantage of making the first move. It is their motion that triggers the escape, and therefore, all of the neural activity that goes into their decision-making and muscle contraction has already occurred by the time the strike begins. Still, it takes the snake’s head about 25 ms to accelerate through the water and reach the fish. There is probably no better stimulus for initiating a C-start than the shock wave generated by the explosive strike of a snake. As a result, the two behaviors—the strike and the C-start—are intertwined. To deal with this truism, tentacled snakes have evolved behaviors that take advantage of escape.

**Fig. 8:**
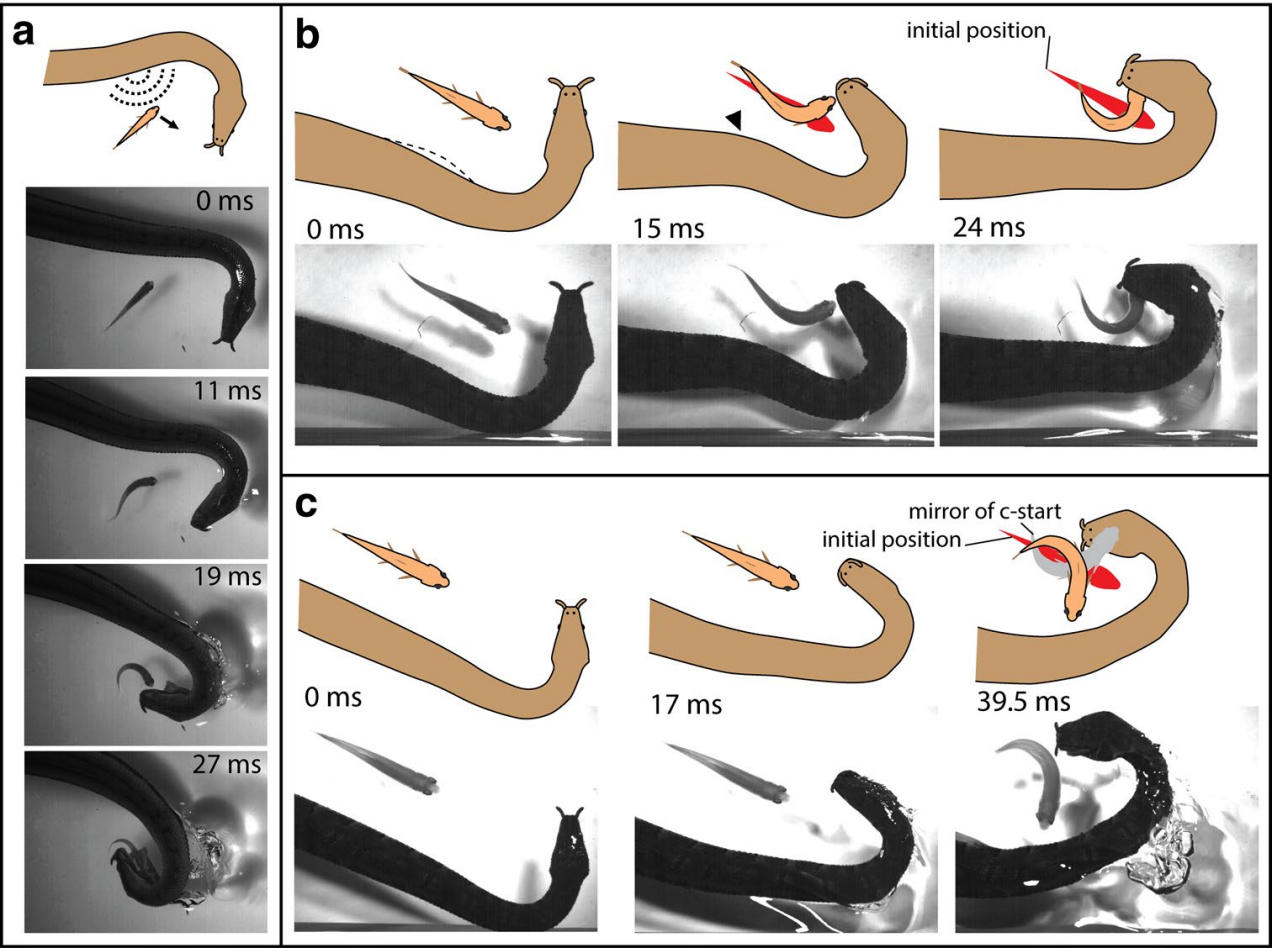
Tentacled snake’s fish trap. **a** When fish approach roughly parallel to the snake’s jaws, movement of the snake’s body precedes the strike, usually triggering the escape response in the wrong direction (for the fish). In this trial, the fish swims directly into the snake’s mouth in less than 30 ms. **b** When fish approach the jaws at a right angle, the body feint usually triggers escape in a predictable direction, and the snake aims for the future location of the escaping fish head. **c** Here, a fish turned toward the body feint and the snake still aimed for the expected (but incorrect) future location of the fish head (*gray outline*)

Careful review of slow motion video revealed that, milliseconds before snakes struck, they moved a portion of their neck on the opposite side of the fish (Fig. 8a). Hydrophone recordings that were coordinated with the high-speed video picked up a pressure wave from this initial movement (Catania 2009). This “body feint” triggered the C-start escape response, but in the wrong direction (from the fish perspective). About 80% of the time, the fish turned toward the oncoming jaws of their attacker, sometimes, swimming straight into the snake’s mouth at top speed (Fig. 8a and see Catania 2009, 2010 for open access movies). This explained the mystery of disappearing fish and short handling times.

However, things only got more interesting from there. Snakes can only startle fish toward their strike when the fish approaches roughly parallel to the snake’s jaws (Fig. 8a). What happens when fish approach at a right angle to the jaws? Rather than letting these fish pass by, the snakes use the same “body feint” to startle fish. They then strike toward the future position of the escaping fish head (Fig. 8b). The snakes literally predict future prey behavior, based on stereotyped movements of a C-start. It is not possible for the snake to use visual feedback to fine tune the trajectory of predictive strikes, because the strike begins before the fish moves, the snake retracts its eyes during the strike, and the retina cannot process the visual scene quickly enough. In case those arguments are not convincing, during some strikes the fish turned toward the “body feint”, instead of away. In those trials, the snake still struck for the predicted (but wrong) future location of the fish’s head. Mirroring the C-start for those trials (Fig. 8c) reveals the snake’s prediction, had the body feint worked.

Do tentacled snakes learn to predict fish movements, or are they born with this ability? This question is philosophically interesting, because striking tentacled snakes presumably never see a C-start. They sit cryptically waiting for a fish to get close, and then strike, but the fish is never where it appeared to be. Instead, in nearly every case, the fish will have moved during the C-start elicited by the strike. This is reminiscent of experiments using vision-shifting goggles that displace the light path by a fixed angle. In a sense, the snake can only make contact with a fish through the prism of a C-start.

**Fig. 9:**
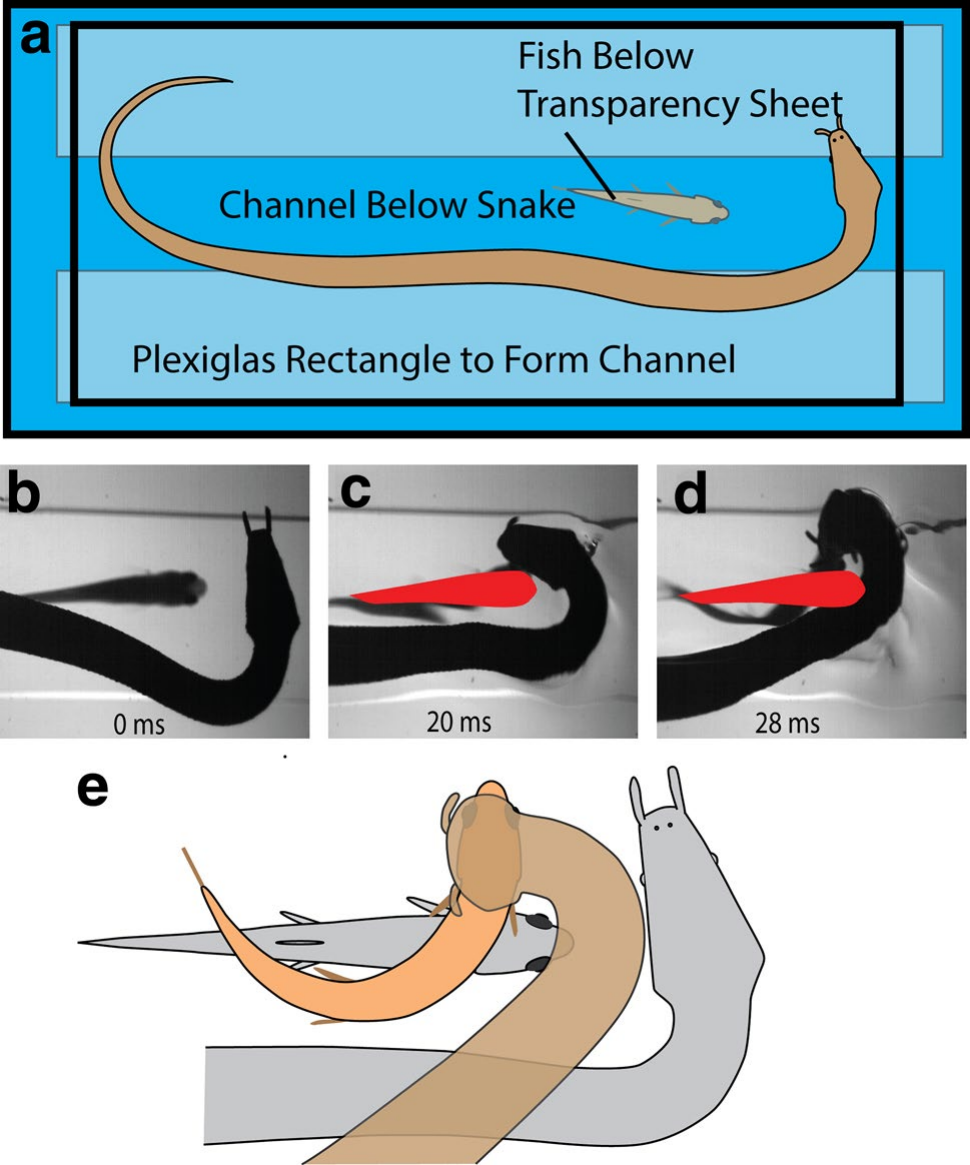
Paradigm and results for investigating predictive strikes in newborn, naïve snakes. **a** To prevent learning during trials, the snakes were above the fish, separated from the fish by a thin transparent barrier that did not prevent C-start responses. **b**–**d** Example of a downwardly directed, predictive strike by a naïve snake. All of ten newborn snakes made predictive strikes. **e** Summary of the spatial relationships between fish and snake for one class of predictive strikes by newborns. *Gray* represents prestrike position, colored represents the average position of the snake, and fish heads for 21 strikes (see Catania 2010 for details and movies)

Determining whether learning was involved required newborn tentacled snakes that had never hunted, (tentacled snakes give live birth). When one of the snakes became gravid, it was strictly monitored when fed, so the newborns could never encounter a fish. When the snake finally gave birth, the hungry, naïve juveniles were tested. The challenge was to collect multiple trials from each snake, without allowing them to ever catch a fish (which could allow learning about C-starts). This was accomplished by separating the fish from the snakes with a thin transparency sheet that did not block the pressure wave from the snake’s body feint, but prevented the snake from contacting the fish (Fig. 9a). The results were clear and compelling; tentacled snakes are born with the ability to predict future prey behavior. They consistently struck at the future position of escaping fish. Moreover, the paradigm revealed a new class of predictive strikes, during which the snake aims downward toward the future position of the head (Fig. 9 and see Catania 2010 for movies). Replacing the thin barrier with a piece of glass prevented the pressure wave from reaching the fish. This last modification prevented C-starts in response to the snake, and (as was the case for C-starts toward the body feint described previously) confirmed that newborn snakes did not “follow” the C-start. Rather they struck toward the expected future location of the fish head, regardless of fish movements. Finally, the lack of C-starts during the glass plate trials emphasizes the role of fish ears and the lateral line system, rather than vision, for triggering C-starts.

These results are a testament to evolution’s ability to shape innate behaviors when the environment provides a reliable framework. In this case, the framework is another innate behavior, the fish C-start. The results also provide another compelling example of Dawkin’s “Rare Enemy Effect”. Presumably, uncommon tentacled snakes exert little selective pressure on diverse fish populations subject to a myriad of more common predators.

What about the mysterious tentacles that inspired the studies in the first place? They were investigated as originally planned (Catania et al. 2010) and found to be densely innervated, water motion sensors that map into the optic tectum in approximate register with vision. They likely provide additional cues to fish position, especially in darkness. The latter results provide a new example of sensory integration in the optic tectum.

## Conclusions

In describing this series of studies, I have attempted to highlight the power of the neuroethological approach. In a world full of species with habitats and senses foreign to the investigator, it is often difficult or impossible to predict the stimuli most relevant to a particular species or the significance of specializations in central and peripheral nervous systems. Experiments that probe the behavioral dimension, key to the field of neuroethology, are often the binoculars that bring experiments into focus. And like binoculars in the field, attention drawn to the commonplace may reveal something unexpected nearby. In my case, an anatomical study of the mole’s star revealed the clues that led to a unique neocortex. However, the key to the cortical maps was behavior. Behavioral experiments aimed at common mole somatosensation revealed exquisite olfaction, helping to explain why earthworms evolved to flee aboveground when moles approach. Finally, tentacled snake behavior is best understood not from the perspective of its own brain, but rather from the brain of its prey.
